# Alois Alzheimer and vascular brain disease: Arteriosclerotic atrophy of the brain

**DOI:** 10.1590/S1980-57642015DN91000013

**Published:** 2015

**Authors:** Eliasz Engelhardt, Lea T. Grinberg

**Affiliations:** 1 Full Professor (retired), Cognitive and Behavioral Neurology Unit – Institute of Neurology/Institute of Psychiatry – Federal University of Rio de Janeiro (UFRJ), Rio de Janeiro-RJ, Brazil.; 2 Assistant Professor, Department of Neurology and Pathology, University of California, San Francisco, San Francisco, USA; 3 Professora Doutora, Departamento de Patologia, Faculdade de Medicina da Universidade de São Paulo, Sao Paulo, SP, Brazil.

**Keywords:** Alzheimer, brain vascular disease, arteriosclerosis, syphilis, Arteriosclerotic atrophy of the brain

## Abstract

Alois Alzheimer is best known for his description of neurofibrillary changes in brain neurons of a demented patient, identifying a novel disease, soon named after him by Kraepelin. However, the range of his studies was broad, including vascular brain diseases, published between 1894 and 1902. Alzheimer described the clinical picture of Arteriosclerotic atrophy of the brain, differentiating it from other similar disorders. He stated that autopsy allowed pathological distinction between arteriosclerosis and syphilis, thereby achieving some of his objectives of segregating disorders and separating them from syphilis. His studies contributed greatly to establishing the key information on vascular brain diseases, predating the present state of knowledge on the issue, while providing early descriptions of what would be later regarded as the dimensional presentation of the now called "Vascular cognitive impairment", constituted by a spectrum that includes a stage of "Vascular cognitive impairment not dementia" and another of "Vascular dementia".

## Abstract (pt)

Alois Alzheimer é conhecido principalmente pela sua descrição de alterações neurofibrilares em neurônios cerebrais de uma paciente demenciada, identificando uma nova doença logo denominada com seu nome por Kraepelin. Entretanto, o âmbito de seus estudos foi amplo, incluindo doenças vasculares cerebrais, publicados entre 1894 e 1902. Alzheimer descreveu o quadro clínico da Atrofia arteriosclerótica do cérebro, diferenciando-a de outros transtornos semelhantes. Ele afirmou que a autópsia permitia distinguir patologicamente entre arteriosclerose e sífilis, alcançando alguns de seus objetivos, segregar transtornos e separá-los da sífilis. Seus estudos contribuíram fortemente para estabelecer as informações-chave sobre doença vascular cerebral, que antecederam o estado do conhecimento atual sobre o assunto, e antecipando o que mais tarde seria visto como a apresentação dimensional do atualmente denominado "Comprometimento cognitivo vascular", constituído por um espectro que inclui um estágio de "Comprometimento cognitivo vascular não demência" e outro, a "Demência vascular".

## INTRODUCTION

**Figure 1:**
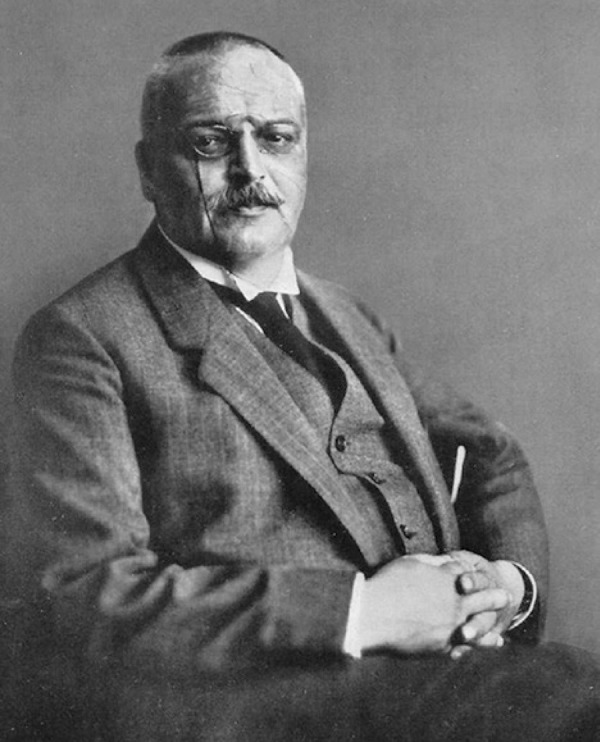
Aloysius (Alois) Alzheimer (1864-1915).

Aloysius (Alois) Alzheimer (1864-1915) (Figure 1), a German psychiatrist and neuropathologist, is best known for his description of the "neurofibrillary tangles", identifying a new disease.^1^ A short time later this disorder was named after him, receiving the denomination of Alzheimer's disease.^2^ Numerous authors wrote about Alzheimer's remarkable discovery. However, the range of his studies was broad and noteworthy, including syphilis, epilepsy, alcoholism, vascular brain diseases, among others. The vascular affections of the brain were intensively studied, and appeared as a series of conferences, lectures and papers published between 1894 and 1902. However, although at the end of each presentation there were image demonstrations, these were not available in the publications. His works contributed greatly to establishing key information for the present state of knowledge on the subject - Vascular dementia and related issues. Alzheimer was not the only researcher to study this theme, but was the one that produced the most comprehensive concepts, clinical descriptions and pathological analysis of vascular brain disease. He cited and commented on important works of renowned colleagues of his time who were also dedicated to this question, and from whom he also drew and incorporated some concepts, such as Durand-Fardel (1854), Forel (1877), Klippel (1892), Binswanger (1894), Campbell (1894), Jacobsohn (1895), Noetzli (1895), Beyer (1896), Windscheid (1902), among others.

The definition of dementia at the time was not entirely clear, including heterogeneous conditions such as General Paralysis of the Insane, Involutional melancholia, Mania, Senile dementia, psychic disorders of the senium, vascular brain diseases, and others, which were scrutinized with the aim of identifying an organic etiology for these so-called "organic psychoses", as well as segmenting them into individual conditions.^3^ It is important to remember that the most well-known causes were syphilis, arteriosclerosis, and senility.^4^ The main concern at the time was to distinguish the several kinds of manifestations from syphilis, a condition manifested as *Dementia paralytica* (Paralytic dementia, General paralysis of the insane). The disorder, known since the 16^th^ century, was later cited by several famous authors. However, it was only in 1822 that Bayle gave the first account of General Paresis of the Insane, describing it as a distinct disease in his medical thesis, with clinical details and pathological aspects. Further knowledge on the disease emerged in the decades that followed, until the discovery of the syphilitic spirochete.^5,6^ Alzheimer was one of the scholars who exposed these concerns. Thus, he always highlighted the characteristic clinical manifestations of Paralytic dementia as items for differential diagnosis (ideas of grandiosity, loss of awareness of the disease, disorganized behavior, restlessness, and other manifestations, as described by Bayle, besides early loss of pupillary reaction to light, progressive gait difficulty, etc.), usually not observed in the arteriosclerotic disease types, or only present late in the course of the illness.^5,7,8^

Reading the entire series of papers on vascular diseases of the brain it is clear that two conditions were emphasized by Alzheimer, the "Arteriosclerotic atrophy of the brain", and the long-standing "Senile dementia". He also wrote extensively about subforms, a collection of diffuse and focal vascular disorders.^7,9^

The aim of the present paper was to focus on Alzheimer's Arteriosclerotic atrophy of the brain, the first of the series, to analyze his thoughts on vascular brain disease, as well as comments on the production of personalities he cited, and that enriched his writings.

## ARTERIOSCLEROTIC ATROPHY OF THE BRAIN

The *Arteriosklerotische atrophie des Gehirns* (Arteriosclerotic atrophy of the brain) (1894) was the first Alzheimer's paper on this disorder, where he described its clinical and pathological findings in a concise publication, further expanded in subsequent papers, emphasizing differences to General paralysis (or Paralysis), one of his aims.^7^ He acknowledged that Klippel, in 1892, France, was the first to describe a comparable condition, the *Pseudoparalysie générale arthritique* (General arthritic pseudoparalysis - *arthritische Pseudoparalysie*).^10^ According to Alzheimer, those who read Klippel's work were left in no doubt that this disorder was coincident with the condition later named in Germany as *arteriosclerotische Hirnatrophy*. However, according to German traditional medicine, atheromatous and arthritic were not coinciding notions as in the French disseminated conception. Thus, probably for this reason Klippel's work in Germany drew less attention and understanding. In Germany, Binswanger and Alzheimer were the first to describe in detail the *arteriosclerotische Hirnatrophie* (Arteriosclerotic brain atrophy), presented at the German Meeting of Psychiatrists, in Dresden, in 1894, where they emphasized the need for its segregation from Paralysis.^9^

Alzheimer stated that the clinical picture of his "common form" of Arteriosclerotic atrophy of the brain initially resembled what he called the "Nervous form". He explained that the disease had an insidious onset, with mild tiredness, headache, dizziness, decrease in sleep, followed by severe irritability and memory deficit. Such cases, rare in institutions, were frequently seen in clinical practice (see also Box 1). Alternatively, a sudden onset with an apoplectiform attack and one-sided paralysis could initiate the picture. He observed that the disorder evolved with an increasing memory impairment and judgment failure, besides abrupt mood swings, stubbornness and childlike behavior. An apparent outer tranquility, orderly attitude, and generally good reasoning clearly distinguished such patients from others with Paralysis. He considered neurological manifestations helpful for the diagnosis and stated that tremor, weakness of the limbs and increased tendon reflexes were typically the first physical symptoms. Additionally, hemiparetic manifestations and speech disturbances, different from paralytic types, were also often present while pupillary reaction changes appeared only in later phases. The terminal stage was characterized by a deep apathetic dementia (*Blödsinn*) or a complete erasing of the memory. Despite this unfortunate situation the patient exhibited quiet and orderly behavior.^7^

**Box 1:** Arteriosclerotic atrophy of the brain: the 1^st^ group or mildest form of arteriosclerosis.^9,12^

This group corresponds to the mildest form of Alzheimer’s named the *Nervöse Form der Arteriosklerose* (Nervous form of arteriosclerosis), according to Windscheid’s 1901 specification. The latter based the diagnosis of the arteriosclerotic process on examinations of the peripheral arteries, and from this he inferred the status of the brain arteries, which he related to mental manifestations, psychic and physical fatigue, headache, dizzy spells and memory impairment, Windscheid’s view of a symptom-complex characteristic of an *Arteriosklerosis cerebri* (Cerebral arteriosclerosis).^13^
Alzheimer, additionally, described that patients often become irritable, incapable of sustained work or continuing their professional tasks. Mental activity appeared to be only possible in deep-rooted paths, mental productivity declined and attention was poorly sustained. The course had a slowly progressive nature, prevailing with symptoms on the threshold of the “Nervous form” range. Such cases, rare in institutions, were frequently seen in clinical practice, as Windscheid recently observed.^9^
Alzheimer described that sometimes patients complained greatly about the memory impairments, not demonstrable with the usual methods, constituting more a subjective feeling of difficulty recalling single facts than a failure as such. Patients could eventually manage, by indirect means, to recover memories initially inaccessible. The least stabilized memories, such as names and numbers, were more vulnerable, as were some older reminiscences. Insight was maintained and the fear of becoming foolish (Blödsinnig) was often expressed. The symptoms were generally prone to regression, and could remain stable for years. In other cases the disease advanced to a severe progressive form.^9^

The postmortem was described by Alzheimer in detail. He considered the macroscopic findings of the brain highly typical, with a mildly cloudy pia, a slightly atrophic cerebral cortex, changes in the cerebral vessels up to the smallest branches, and the white matter traversed by yellowish streaks, which appeared as vessel lacunas, whose surrounding tissues were sometimes considerably indurated. Around most vessels he found wide spaces filled with fluid, particularly in the basal ganglia and the internal capsule. The microscopic examination revealed, dispersed in the cortex, numerous small aneurysms, capillary bleeding, focal condensations of the neuroglia, and accumulation of granulated cells. Small softenings eventually appeared where the latter were greatly augmented.^7^ The presence of severe arteriosclerotic changes of the brain vessels and tissue degeneration, apparently directly associated, were noted consistently. These basic changes Alzheimer considered entirely different from those of Paralysis, thus achieving one of his objectives.^7^ Taking into account the arteriosclerotic changes of the brain vessels and the related atrophic findings in the cortex, hemispheric white matter, and basal ganglia, the disease he had described was denominated in 1897 as *Arteriosklerotische Demenz* (Arteriosclerotic dementia) or *Atrophie des Gehirns* (Atrophy of the brain).^11^

Alzheimer, later discriminated two groups of severity of brain arteriosclerosis:^9,12^ the 1^st^ group, constituting the mildest form, he named the Nervous form of arteriosclerosis, according to Windscheid's specification (1901)^13^ (Box 1), and the 2^nd^ group, consisting of Progressive arteriosclerotic brain degeneration, according to Jacobsohn's concept (1895)^14^ (Box 2).

**Box 2:** Arteriosclerotic atrophy of the brain: the 2^nd^ group encompassing “severe progressive arteriosclerotic brain degeneration”.^9,12^

This group encompasses cases of *Progressive arteriosklerotische Hirndegeneration* (Progressive arteriosclerotic brain degeneration) and includes cases related to the “severe form of arteriosclerosis of the central nervous system”, which manifests with multiple softenings, according to Jacobsohn’s concept, based on neuropathology and clinical cases.^14^ Besides arteriosclerotic lesions of the basal ganglia and the medulla oblongata, Jacobsohn’s main interest, multiple bleedings and softenings in the cerebral cortex and hemispheric white matter, among other structures, were described.^3,12^
Here, Alzheimer observed that initial symptoms resembled the “Nervous form”. However, severe psychic symptoms soon appeared, in cases where the disease had not begun with these symptoms. Episodes of irritability, inflexible stubbornness, and also states of uncontrollable restlessness, could also emerge. The interaction with the patient showed only minor real impairments. Attention was severely disturbed, while older memories still retained substantial material, revealed by means of arduous questioning. Interests diminished, but some motivation could improve the patient’s attitude, e.g., a visit from relatives. A mournful depressed mood was often seen. Sensory illusions and delusions can appear. Ideas of grandiosity were not evident. The patient gradually merged into a progressively deeper and blunter dementia (*Verblödung*), however parts of the former personality remained evident for a long period. Following apoplectic-like attacks, focal manifestations, such as asymbolic behavior, language impairment, visual field changes, cortical movement disturbances, could be observed, and pupils rarely lost reactivity. Attacks could also manifest solely in the psychic domain, such as transient states of stupor, perplexity, hallucinatory excitation states, and disorientation with maniacal excitation. Awareness of the disease was clearly maintained for a long period.^9^

## COMMENTARIES

Alzheimer described the clinical picture of Arteriosclerotic atrophy of the brain or Arteriosclerotic brain atrophy, distinguishing these from other similar disorders, clinically and pathologically. He stated that autopsy permitted differentiation between an arteriosclerotic cause and syphilis. Thus, he reached some of his proposed aims, namely, to segregate conditions and separate them from syphilitic brain disease. He further subdivided the disorder into two groups, the first of which he named the "Nervous form" with mild symptoms which could remain stable or even regress. The disease could advance to a severe progressive form. The other group pools cases of "Severe progressive arteriosclerotic brain degeneration", that could sometimes begin in the same manner as the "Nervous form" but in which severe psychic occurrences soon ensued, with later deeper impairments, gradually merging into a progressively profounder and blunter dementia.^9^

It is possible to recognize in these two groups, the precursor of what would later be distinguished as the "Vascular cognitive impairment" spectrum, with a "Vascular cognitive impairment not dementia" and another of "Vascular dementia" stage.^15,16^ (see also Box 3).

**Box 3:** Vascular cognitive impairment – present and past.^3,9,15,16,19^

The term “Vascular cognitive impairment” refers to the cognitive impairment due to cerebrovascular disease, encompassing all levels of cognitive decline, from the brain-at risk stage, passing across an intermediate not-dementia phase, and finally ending in the plainly expressed “Vascular dementia”.^15,16^
Among the several types of vascular lesion that may be found, one of the most frequent is subcortical white matter affection (white matter atrophy), due to chronic cerebral ischemia, described by Binswanger, and acknowledged by Alzheimer, as a “subform of arteriosclerotic brain atrophy”. Alzheimer provided a separate description of this subform, resulting from his own observations, and where the autopsy showed a “severe arteriosclerotic disease of the long vessels of the deep white matter” with highly atrophic hemispheric white matter.^3,9,19^

Several authors have published interesting analyses on some of Alzheimer's papers. In 1991, Förstl and Howard focused only on Alzheimer's 1898 paper, whose main focus is Senile dementia and also comments on some of the subforms.^17^ Mast et al. analyzed Alzheimer's (and Binswanger's) vascular studies, commenting on Alzheimer's findings and emphasizing the relevance of white matter lesions.^18^ Remarks on this subject were also presented by Román, in his 1999 review on the history of dementia. He mainly cites Alzheimer's papers of 1898 and 1902, and focused on Arteriosclerotic brain degeneration, transcribing and commenting on its clinical and pathological aspects, and also briefly remarking on some subforms.^19^

## CONCLUSION

Alzheimer undertook the task of differentiating apparently similar arteriosclerotic brain disorders, distinguishing between them and with syphilitic brain disease. These objectives were reached after laborious work expressed in a detailed manner in his publications.
